# A Small Conductance Calcium-Activated K^+^ Channel in *C. elegans*, KCNL-2, Plays a Role in the Regulation of the Rate of Egg-Laying

**DOI:** 10.1371/journal.pone.0075869

**Published:** 2013-09-10

**Authors:** Cavita K. Chotoo, Gary A. Silverman, Daniel C. Devor, Cliff J. Luke

**Affiliations:** 1 Department of Cell Biology, University of Pittsburgh, Pittsburgh, Pennsylvania, United States of America; 2 Department of Pediatrics, University of Pittsburgh, Children’s Hospital of Pittsburgh of UPMC and Magee-Women’s Hospital Research Institute, Pittsburgh, Pennsylvania, United States of America; Virginia Commonwealth University, United States of America

## Abstract

In the nervous system of mice, small conductance calcium-activated potassium (SK) channels function to regulate neuronal excitability through the generation of a component of the medium afterhyperpolarization that follows action potentials. In humans, irregular action potential firing frequency underlies diseases such as ataxia, epilepsy, schizophrenia and Parkinson’s disease. Due to the complexity of studying protein function in the mammalian nervous system, we sought to characterize an SK channel homologue, KCNL-2, in *C. elegans*, a genetically tractable system in which the lineage of individual neurons was mapped from their early developmental stages. Sequence analysis of the KCNL-2 protein reveals that the six transmembrane domains, the potassium-selective pore and the calmodulin binding domain are highly conserved with the mammalian homologues. We used widefield and confocal fluorescent imaging to show that a fusion construct of KCNL-2 with GFP in transgenic lines is expressed in the nervous system of *C. elegans*. We also show that a KCNL-2 null strain, *kcnl-2*(*tm1885*), demonstrates a mild egg-laying defective phenotype, a phenotype that is rescued in a KCNL-2-dependent manner. Conversely, we show that transgenic lines that overexpress KCNL-2 demonstrate a hyperactive egg-laying phenotype. In this study, we show that the vulva of transgenic hermaphrodites is highly innervated by neuronal processes and by the VC4 and VC5 neurons that express GFP-tagged KCNL-2. We propose that KCNL-2 functions in the nervous system of *C. elegans* to regulate the rate of egg-laying.

## Introduction

In mammals, four KCNN genes that encode the small- (K_Ca_2.1, K_Ca_2.2, K_Ca_2.3) and intermediate-conductance (K_Ca_3.1) Ca^2+^-activated potassium channels have been cloned [1–3]. SK channels have a diverse expression profile and function to hyperpolarize cells in which they are expressed. In the nervous system, the SK channel family functions to generate a component of the afterhyperpolarization (AHP) that follows action potentials [4]. This AHP is the rate-limiting step in preserving precision and frequency of pacemaker activity in neurons. SK channels have been shown to regulate the excitability of Purkinje cells, hippocampal pyramidal neurons and dopaminergic neurons, and as a result SK channels have been implicated in a wide array of neurological disorders including episodic ataxia type 2, epilepsy and schizophrenia [5–9]. The biological functions of SK channels have been widely investigated using mouse models. A K_Ca_2.2 deletion mutant, the frissonant mouse model, displays altered AHPs that result in irregular action potential firing frequency leading to rapid tremor and locomotor instability [10]. Another mouse model in which K_Ca_2.1 and K_Ca_2.2 channel expression was specifically knocked down in the deep cerebellar nuclei showed phenotypes reminiscent of cerebellar ataxia deficits, such as postural instability, defective motor learning and severe incoordination [5]. However, despite the evidence that shows the role of SK channels in the central nervous system (CNS), as demonstrated in transgenic mouse lines, the correlation of SK channel function to the described neurological disorders has not been ascertained.

In addition to its role in the nervous system, SK channels are a potential pharmacological target for hypertension and incontinence due to their functions in regulating the contractility of vascular and urinary bladder smooth muscle cells, respectively [11–14]. Additionally, K_Ca_2.3-overexpression in mice has been shown to hinder uterine contractions during labor resulting in the death of the parent and pups, while suppression of K_Ca_2.3 did not result in phenotypic consequences [15].

*C. elegans* is a particularly advantageous model organism to study SK channel function due to its molecular and genetic tractability as well as its anatomical simplicity. As the cuticle and embryo eggshell are transparent, cellular processes can be visualized *in vivo* by widefield and confocal microscopy. The nervous system of *C. elegans* has been mapped from its early developmental stages throughout adulthood and this information can afford the study of SK channel function in isolated neuronal circuits [16]. Mutations that render the channel as inactive or hyperactive often result in opposing phenotypes and therefore study of the behavior of *C. elegans* can yield physiological knowledge of SK channels. In *C. elegans* there are four genes, *kcnl-1* through *-4*, that encode SK channel homologues. Herein, we demonstrate the biological significance of the KCNL-2 protein. We show that KCNL-2-expression is evident in the nervous system of *C. elegans* and that KCNL-2 plays a novel role in the regulation of the rate of egg-laying which is likely dictated through its expression in the nervous system.

## Results

### Essential Structural Domains of Mammalian SK Channels are Conserved in KCNL-2

**Figure 1:**
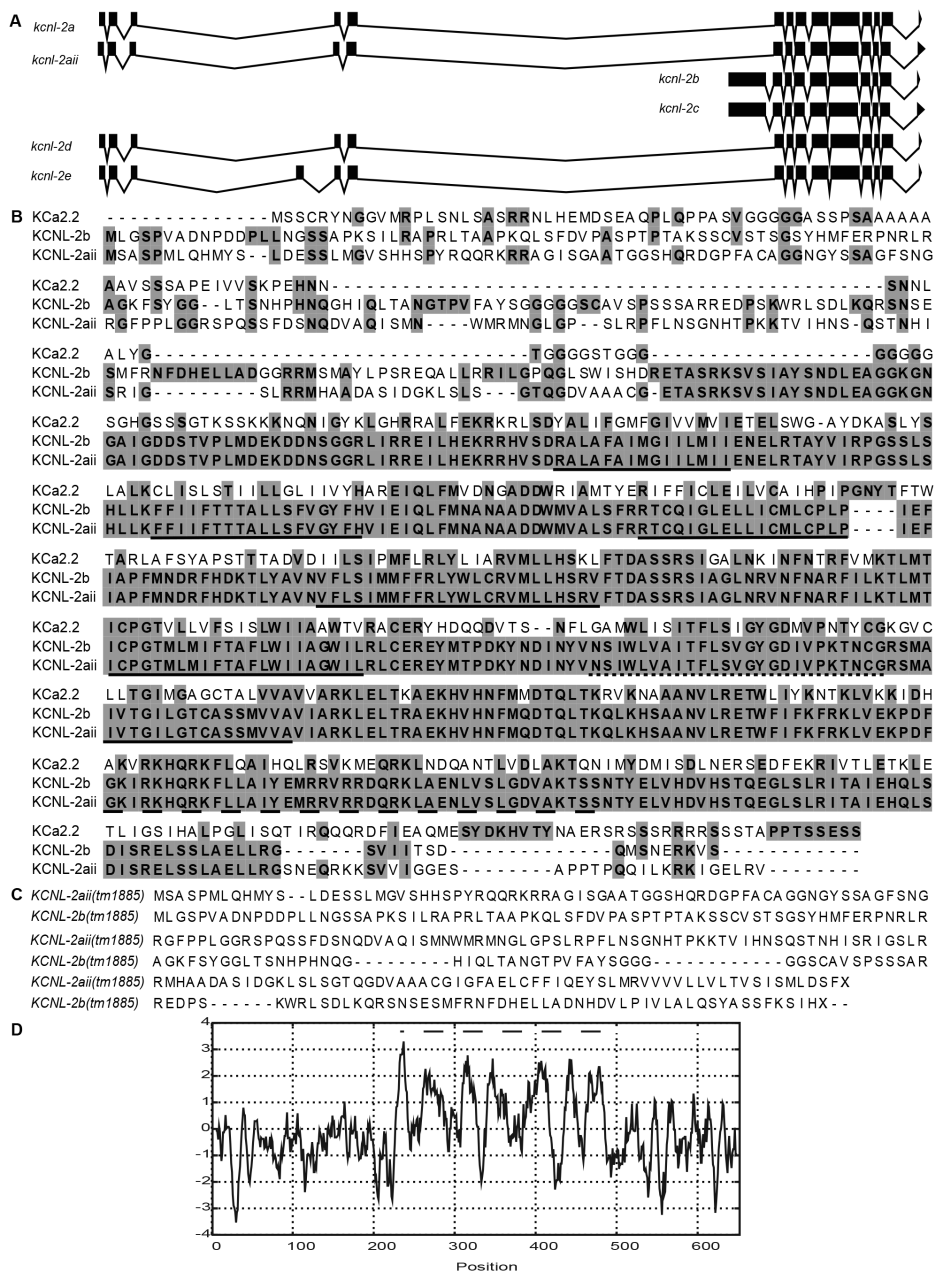
Structural analysis of KCNL-2 isoforms. A. The WRM063DE08 fosmid encodes six isoforms of KCNL-2, which vary in their amino and carboxy termini of the predicted protein structure. The exon structures show that there are two initiation sites that are 9.5 kb apart and two stop codons that are 68 bp apart. B) KCNL-2 isoforms have 37% identity with K _Ca_2.2, while KCNL-2-aii and -b are 71% identical. The greatest degree of conservation occurs in the core of the channel where the S1-S6 transmembrane domains (underlined solid), the potassium-selective pore filter (underlined dotted), and the calmodulin binding domain (underlined dashed) are encoded. C) *kcnl-2*(tm1885), a 962 bp deletion, results in a premature stop codon before the first TMD of KCNL-2-aii and -b. D) Kyte-Doolittle hydropathy plot of the KCNL-2-a isoform showing seven domains that are highly hydrophobic, which represent the 6 TMDs and the pore motif (dashed line).

The *C. elegans* genome contains 80 potassium channel-encoding genes whose products serve to regulate the excitability of cells and their residing organ systems [17]. Of these, four genes, *kcnl-1* (coding sequence (CDS) B0399.1), *kcnl-2* (CDS F08A10.1), *kcnl-3* (CDS C03F11.1) and *kcnl-4* (CDS C53A5.5), encode channels that are homologous to human SK channels. Up to eight splice variants (a through h) of *kcnl-2* have been predicted based on expressed sequence tag (EST) data (http://www.wormbase.org, WS231, 07.12.2012). The splice variants *kcnl-2-f*, -*g* and -*h* (accession numbers NM_001263764, NM_001263763 & NM_001263765, respectively) were identified more recently and their analysis remains outside the scope of this study since they were not encoded by the WRM063DE08 fosmid (*C. elegans* Reverse Genetics Core Facility, Canada) that was used for our investigation of KCNL-2. We sequenced and analyzed the yk1015a02, yk1103e07, yk1295d10 and yk1401h05 cDNA clones (obtained from Yuji Kohara, National Institute of Genetics of Japan) encoding transcripts of *kcnl-2*. Sequencing of yk1295d10 shows a full-length clone of *kcnl-2b* (accession number NM_181991) that is incompletely spliced, while the sequence of yk1401h05 reveals an alternative isoform of *kcnl-2a* (accession number NM_059833), which we termed *kcnl-2aii* (accession number JX455834). yk1103e07 and yk1015a02 encode partial transcripts of *kcnl-2-c* and *-d*, respectively (accession numbers NM_181991 & NM_181992). In this study, we analyzed the expression of the first five isoforms of KCNL-2 encoded by the fosmid in addition to the newly identified KCNL-2aii isoform as depicted in Figure 1A. The a, aii, d, and e splice variants share the same initiation site, are 98% identical and their open reading frames span ~12.5 kb, while the b and c splice variants have the same initiation site, are 98% identical, but their exons span ~3 kb (Figure 1A). Additionally, as shown in Figure 1A, the a, b, d and e splice variants share a common stop codon, while the aii and c splice variants are alternatively spliced and share a second stop codon. Alignments of the peptide sequences of the KCNL-2 isoforms show that the worm orthologue bears 37% identity to K_Ca_2.2 (Figure 1B), and 35% identity to the K_Ca_2.1, K_Ca_2.3 and K_Ca_3.1 channels. The greatest degree of homology occurs in the core of the channel (~50% identity) where the six transmembrane domains (Figure 1B, solid lines), the potassium-selective pore (Figure 1B, dotted line) and the calmodulin binding domain (Figure 1B, dashed line) are conserved. There is little homology between the amino and carboxy termini of KCNL-2 and mammalian SK channels. Hydrophobicity scores of the primary sequence of KCNL-2a reveal seven highly hydrophobic regions corresponding to the S1-S6 transmembrane domains and the pore motif (Figure 1D) [18]. Since the structural domains that are known to be important in mammalian SK channels are conserved, this suggests that KCNL-2 would have similar biological functions to its mammalian homologues. A KCNL-2 deletion mutant, *kcnl-2*(*tm1885*), was generated by the National Bioresource Project (Japan). As shown in Figure 1C, the *kcnl-2*(*tm1885*) mutation results in a frameshift that leads to a premature stop codon upstream of the first transmembrane domain of all isoforms of KCNL-2. Therefore, the *kcnl-2*(*tm1885*) mutation likely results in a null channel.

### Phenotypic Analysis of KCNL-2

**Figure 2:**
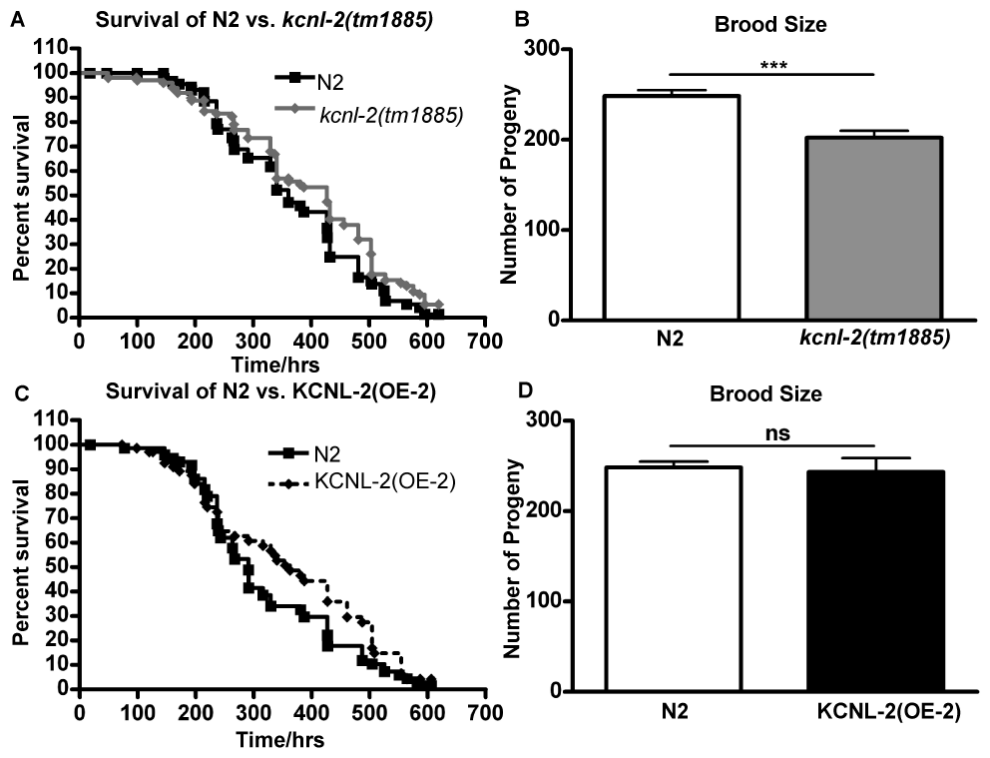
Phenotypic analysis of *kcnl-2*(*tm1885*) and KCNL-2(OE), a transgenic line that overexpresses *p*_*kcnl-2*_ *kcnl-2*(*taa2*)*::gfp* in the N2 background. A,C. Kaplan-Meier Survival Curves showing the longevities of N2 vs. *kcnl-2*(tm1885) (n=80; p=0.06, Logrank test) or N2 vs. KCNL-2(OE-2) (n=50; p=0.12, Logrank test). B,D. Brood size of N2 vs. *kcnl-2*(tm1885) (n≥17; p<0.001, Student’s *t* test) or N2 vs KCNL-2(OE-2) (n≥13; p=0.77, Student’s *t* test).

Given the structural similarity of KCNL-2 to mammalian SK channels and the well-defined anatomy of *C. elegans*, we propose that using *C. elegans* would serve as a practical model system to further understand the physiology of SK channels. In order to investigate possible functions of KCNL-2, we carried out phenotypic assays on *kcnl-2*(*tm1885*) and KCNL-2-overexpressing transgenic lines at 20^o^C. The *kcnl-2*(*tm1885*) strain was outcrossed 8 times to remove extraneous mutations that resulted from TMP treatment. To monitor the overall health of these organisms that likely lacked the KCNL-2 channel, we assayed the survival of a population of late L4 animals from the described worm strains. Figure 2A shows the Kaplan-Meier survival curves of N2 and *kcnl-2*(*tm1885*) worms. Based on a Logrank test, the longevities of N2 vs. *kcnl-2*(*tm1885*) organisms are not significantly different suggesting that the KCNL-2 channel is not required for survival of the adult animals. To examine possible functions of KCNL-2 in reproduction, we measured the brood size of *kcnl-2*(*tm1885*) animals. The brood size of *kcnl-2*(*tm1885*) organisms was assessed by counting the number of progeny after 48 hrs of development at 20^o^C. As shown in Figure 2B, the brood size of *kcnl-2*(*tm1885*) organisms is significantly less than the number of progeny produced by N2 animals. This smaller brood size of *kcnl-2*(*tm1885*) animals can be attributed to a population of the progeny undergoing larval arrest and eventual death due to a post-embryonic developmental delay (Figure S1A). Although loss of KCNL-2 led to larval arrest and slow growth, transformation of *kcnl-2*(*tm1885*) organisms with the *kcnl-2* gene (tagged and untagged) did not rescue these phenotypes. The lack of rescue of these phenotypes may be due to extraneous mutations elsewhere in the *kcnl-2*(*tm1885*) genome that were not removed by outcrossing with N2 organisms. To investigate the underlying basis of the post-embryonic developmental delay seen in *kcnl-2*(*tm1885*) organisms, an alternative KCNL-2 knockout strain was obtained from the Caenorhabditis Genetics Center (CGC, University of Minnesota), *kcnl-2*(*ok2818*). This mutation results in the deletion of transmembrane domains 4, 5 and 6 and the K^+^-selective pore region and therefore *kcnl-2*(*ok2818*) is likely to be a KCNL-2 null strain. The *kcnl-2*(*ok2818*) organisms were not developmentally delayed subsequent to outcrossing with N2 animals four times and therefore this phenotype is not specific to the loss of KCNL-2 (Figure S1B).

Since the *kcnl-2*(*tm1885*) animals did not yield an overt phenotype that could be rescued by transforming the null organisms with the *kcnl-2* gene and phenotypic anomalies are often observed in SK knockout mouse models, we sought to determine if overexpression of KCNL-2 in *C. elegans* would yield an overt phenotype. Transgenic lines overexpressing *p*_*kcnl-2*_ *kcnl-2*(*taa2*)*::gfp* in the N2 background (line 2), KCNL-2(OE-2), were assayed for longevity and brood size. Overexpression of the KCNL-2 gene did not result in observable differences in longevity (Figure 2C) and brood size (Figure 2D) when compared to N2 animals. Also, as shown in Figure S1C, no observable differences were seen in the post embryonic development of KCNL-2(OE-2) animals relative to N2 worms. These results demonstrate that overexpression of KCNL-2 did not result in deleterious effects on survival of the organisms or produce defects in gametogenesis and fertilization.

### KCNL-2 is Expressed in the Nervous System

**Figure 3:**
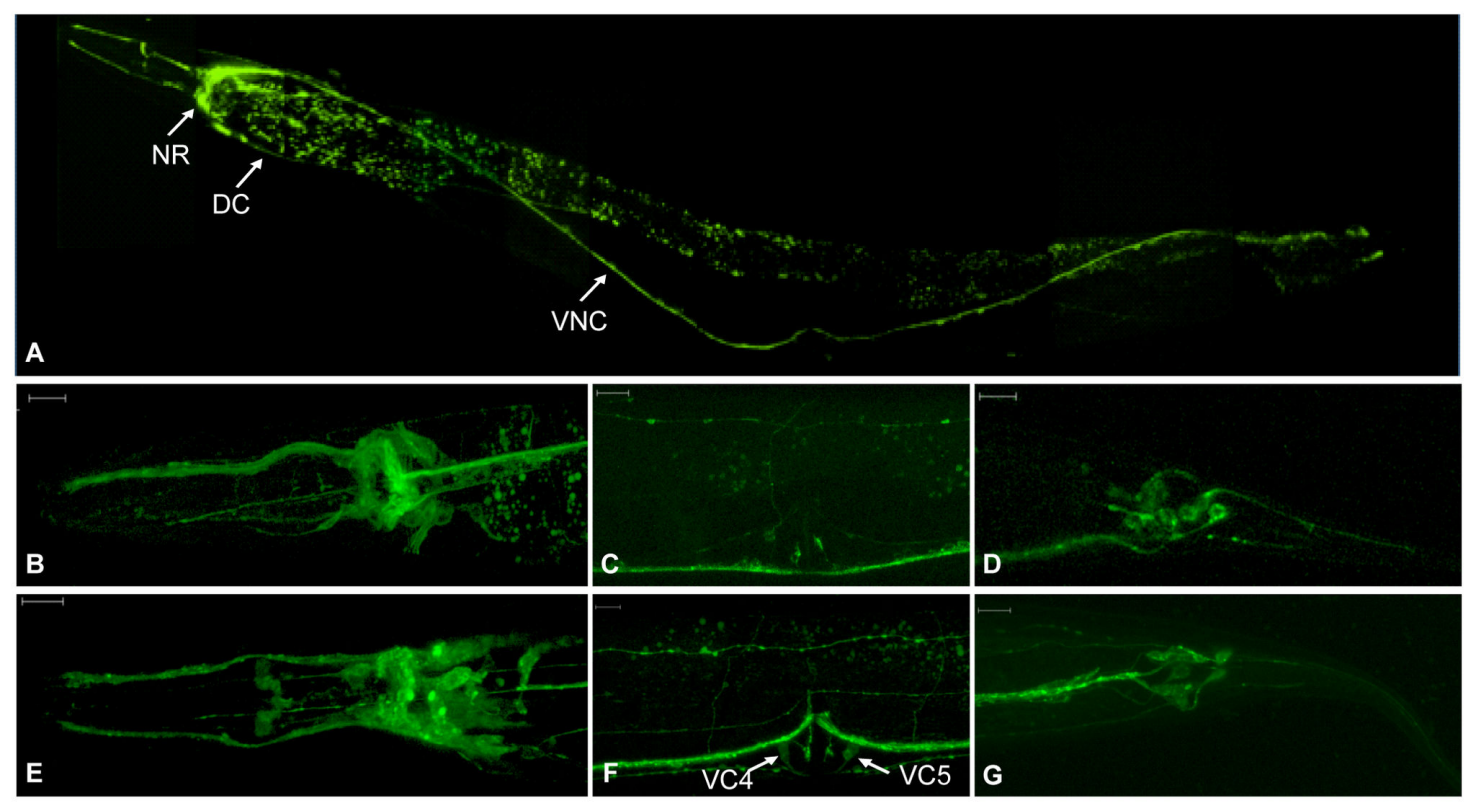
Expression pattern of KCNL-2. A. The *kcnl-2* gene was amplified from the WRM063DE08 fosmid and GFP was fused to the latter stop codon to produce a translational gene fusion that tagged the channel at the carboxy terminus. *p*_*kcnl-2*_ *kcnl-2*(taa2)*::gfp* localized to the neurons of the head, the NR, motor neurons of the VNC, the DC, the pharyngeal nervous system, and to tail ganglia (widefield fluorescent image of strain VK1567). B–G. Magnified confocal images of *p*_*kcnl-2*_ *kcnl-2*(taa2)*::gfp* (B–D) and *p*_*kcnl-2*_ *gfp* :*:*(atg2) *kcnl-2* (E-G; strain VK1401) when expressed in the N2 background (Scale bar=10 µm). B,E. Neurons of the head, the pharyngeal nervous system, and cell bodies of the nerve ring. C. Neuronal processes innervating the vulva. F. Neuronal processes at the vulva, including the VC4 & VC5 cell bodies (arrows). D,G. Tail ganglia.

To aid in the identification of the biological functions of KCNL-2, we sought to determine its localization. KCNL-2 was fused to GFP so that it was effectively tagged at the amino terminus (*p*_*kcnl-2*_ *gfp::kcnl-2*) or at the carboxy terminus (*p*_*kcnl-2*_ *kcnl-2::gfp*). As shown in Figure 1A, the coding sequences for splice variants a, aii, d and e share the same initiation site (atg1), while those of splice variants b and c occur ~9.5 kb downstream of the first initiation site (atg2). Similarly, splice variants a, b, d, and e end at a stop codon (taa1) that is 68 bp upstream of the stop codon of splice variants aii and c (taa2). Therefore, four constructs were made where GFP was fused to either initiation site or stop codon in order to determine if the splice variants are differentially expressed. Expression of the transformation vectors was driven under 1,951 bp of the KCNL-2 endogenous promoter. Confocal fluorescent imaging of transgenic lines expressing these constructs in the N2 background showed a neuronal expression pattern (Figure 3). Figure 3A shows the expression of KCNL-2 when tagged at the latter stop codon (*p*_*kcnl-2*_ *kcnl-2(taa2)::gfp*). As shown in Figure 3A, KCNL-2 is expressed in head neurons, the nerve ring (NR), motor neurons of the ventral nerve cord (VNC), the dorsal cord (DC) and tail ganglia. Figure 3B–D show magnified images of *p*_*kcnl-2*_ *kcnl-2*(*taa2*)*::gfp*, while Figure 3E–G show magnified images of *p*_*kcnl-2*_ *gfp*:*:(atg2) kcnl-2*. It is noteworthy to mention that *p*_*kcnl-2*_ *kcnl-2*(*taa2*)*::gfp* was expressed in many neuronal processes innervating the vulva (Figure 3C), while *p*_*kcnl-2*_ *gfp*:*:*(*atg2*) *kcnl-2* shows expression in the VC4 and VC5 neurons (arrows) of the egg-laying apparatus in addition to other neuronal processes innervating the vulva (Figure 3F). This absolute neuronal expression profile suggests that KCNL-2 in *C. elegans* functions similarly to mammalian SK channels in regulating the excitability of neurons.

**Figure 4:**
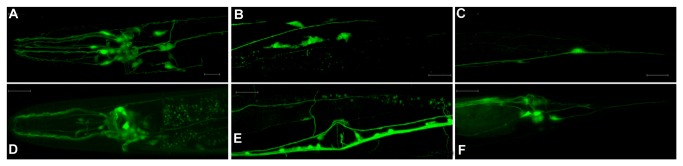
Confocal fluorescent images showing the expression pattern of the KCNL-2 promoter-GFP constructs. A–C. *p*_*kcnl-2*(*atg1*)_ *gfp* is expressed in neurons of the head and NR (A), the vulval muscles (B), and tail ganglia (C) (strain *vk1323*). D–F. *p*_*kcnl-2*(*atg2*)_ *gfp* is expressed in head neurons and NR (D); VNC, VC4 & VC5, and DC (E); and tail ganglia (F) (strain *vk1327*).

Faint protein expression in tissues can be difficult to visualize. Since GFP diffuses freely throughout the cytoplasm of cells in which it is expressed, we made two constructs to drive GFP expression under the KCNL-2 promoter to aid in determining the complete expression profile of KCNL-2 that may be masked in transgenic lines that express GFP-tagged KCNL-2. The first construct, *p*_*kcnl-2*(*atg1*)_ *gfp*, encompassed the coding sequence of GFP which was expressed downstream of the promoter of KCNL-2 (1,951 bp upstream of atg1). The second construct, *p*_*kcnl-2*(*atg2*)_ *gfp*, expressed GFP at the second initiation site where the coding sequences for kcnl-2-b and -c were deleted. As shown in Figure 4A–C, *p* **_*kcnl-2*(*atg1*)_ *gfp* showed a neuronal expression profile with additional GFP-expression in the vulval muscles. As shown in Figure 4D–F, *p* **_*kcnl-2*(*atg2*)_ *gfp* showed a strict neuronal expression profile that complemented the expression profile of the *p*_*kcnl-2*_ *kcnl-2::gfp* constructs. *p*_*kcnl-2*(*atg2*)_ *gfp* readily labels the VC4 and VC5 neurons and displays a highly innervated vulva, a feature that is lacking in the expression profile of *p*_*kcnl-2*(*atg1*)_ *gfp*. Ultimately, since both *p*_*kcnl-2*(*atg1*)_ *gfp* and *p*_*kcnl-2*(*atg2*)_ *gfp* are expressed in different neurons, this suggests that the various isoforms of KCNL-2 may have differing neuronal expression profiles.

### Overexpression of KCNL-2 Causes a Hyperactive Egg-Laying Phenotype

**Figure 5:**
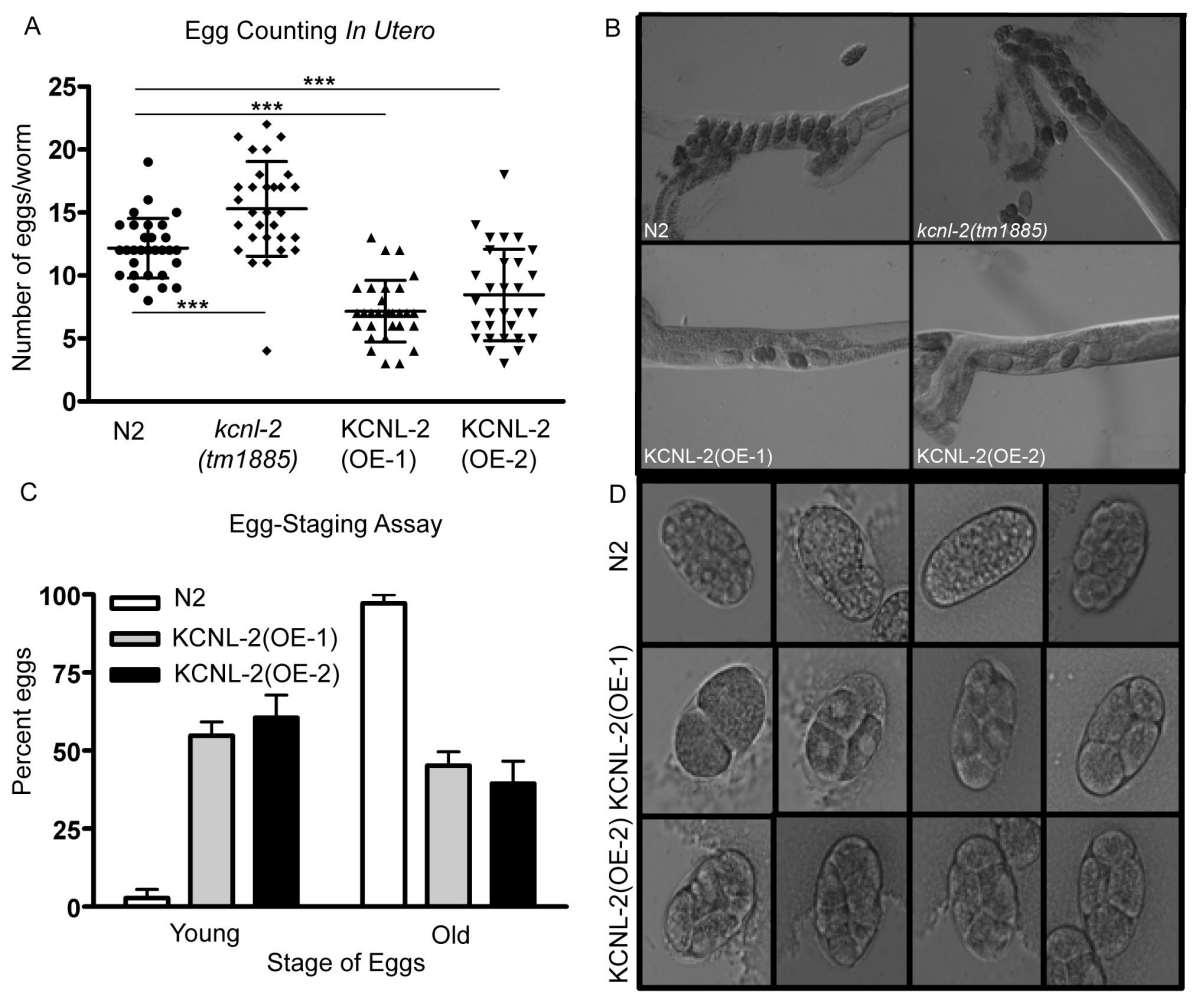
Overexpression of KCNL-2 in the N2 background causes a Hyperactive Egg-Laying Phenotype. A. An unlaid egg assay was carried out by dissolving the cuticle of adult worms in 1.8% sodium hypochlorite and shows that the number of eggs *in utero* in transgenic lines that overexpress KCNL-2 are significantly less than the number of eggs retained *in utero* in N2 and *kcnl-2*(tm1885) strains (p<0.001, Student’s *t* test), while *kcnl-2*(tm1885) has a significantly increased number of eggs retained *in utero* relative to N2 worms (p<0.001, Student’s *t* test). B) Representative images of worms from each strain. C) Egg-staging assays reveal that the proportion of young eggs from KCNL-2(OE-1) (strain VK1000) (54.82±7.65%) and KCNL-2(OE-2) (strain VK1065) (60.57±12.48%) was significantly greater than those of N2 worms (2.78±4.81%) (n=3, p<0.05; Kruskal–Wallis *H* test).

The expression of KCNL-2 in neurons that innervate the vulva may implicate KCNL-2 in the regulation of the rate of egg-laying. To investigate this phenomenon, we carried out unlaid egg assays and egg-staging assays as described previously [19]. Hyperactive or egg-laying constitutive (Egl-C) mutants are characterized as having fewer eggs *in utero* and as laying a greater percent of early-stage eggs relative to wild type animals, while egg-laying defective (Egl) mutants expel their eggs less efficiently and retain a greater number of eggs *in utero* [19–21]. A fertilized egg develops *in utero* for approximately 2.5 to 3 hours in wild-type animals, at which point it has greater than 100 cells under normal developmental conditions. Behavioral assays were conducted on the non-integrated transgenic lines that overexpressed *p*_*kcnl-2*_ *kcnl-2*(*taa1*)*::gfp* and *p*_*kcnl-2*_ *kcnl-2*(*taa2*)*::gfp* in the N2 background, abbreviated KCNL-2(OE-1) and KCNL-2(OE-2) for overexpression lines 1 and 2, respectively. From microinjections, eight independent transgenic lines, VK1000-1003 and VK1065-1068, were obtained when N2 organisms were transformed with 90 ng/µl *p*_*kcnl-2*_ *kcnl-2*(*taa1*)*::gfp* or *p*_*kcnl-2*_ *kcnl-2*(*taa2*)*::gfp*, respectively, and the data is shown for one representative KCNL-2(OE-1) or KCNL-2(OE-2) line. Egg-counting *in utero* or egg-staging assays were conducted on gravid adult hermaphrodites 36 hours after the late larval L4 stage at 20^o^C. As shown in Figure 5A and 5B, the KCNL-2 overexpressing transgenic lines have a significantly lower number of eggs *in utero*, whereas the *kcnl-2*(*tm1885*) animals have a significantly higher number of eggs *in utero* when compared to N2 animals. This phenomenon of fewer eggs retained *in utero* at the adult stage was demonstrated by eight independent transgenic lines as defined in Table S1 (VK1000-1003 and VK1065-1068) and is suggestive of a hyperactive egg-laying phenotype. In both KCNL-2(OE-1) and KCNL-2(OE-2) strains, approximately 50% of the population of animals from 4 transgenic lines had fewer than or equal to 7 eggs retained *in utero* per worm. Egg-staging assays revealed that the proportion of young eggs from KCNL-2(OE-1) (54.8±4.4%) and KCNL-2(OE-2) (60.6±7.2%) was significantly greater than those of N2 animals (2.8±2.8%). Taken together these data suggest that KCNL-2-overexpressing transgenic lines demonstrate a hyperactive egg-laying phenotype.

**Figure 6:**
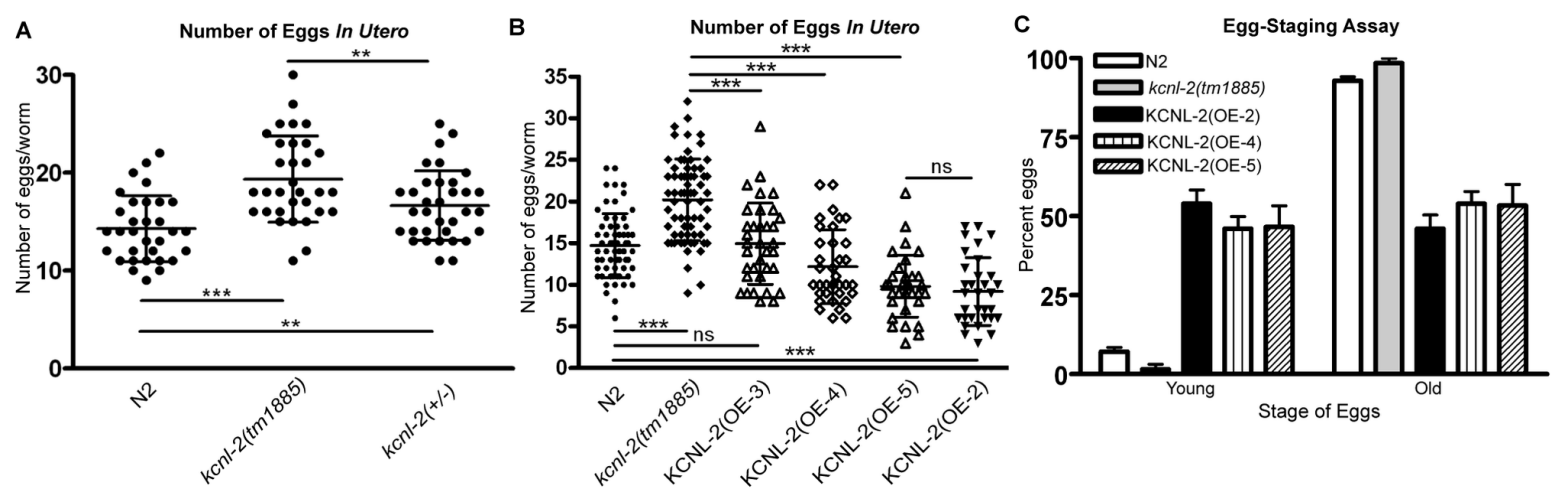
Rescue of the mild Egl phenotype shown by *kcnl-2*(*tm1885*) worms. A. *kcnl-2*(tm1885) animals retain a significantly greater number of eggs *in utero* relative to N2 animals (P<0.0001). The number of eggs *in utero* in heterozygotes (*kcnl-2*(-/+)) is significantly greater than that of the N2 animals (p=0.007), but significantly less than the average number of eggs found in *kcnl-2*(tm1885) animals (p=0.008). B. Transformation of *kcnl-2*(tm1885) with 1 ng/µl, 10 ng/µl or 90 ng/µl of *p*_*kcnl-2*_ *kcnl-2*(taa2)*::gfp* gives the transgenic lines KCNL-2(OE-3) (strain VK2220), KCNL-2(OE-4) (strain VK1004) and KCNL-2(OE-5) (strain VK1041), respectively. The average number of eggs retained *in utero* by KCNL-2(OE-3) was not significantly different from the N2 animals (P=0.8) but was significantly different from the *kcnl-2*(tm1885) organisms (p<0.0001). KCNL-2(OE-4) and KCNL-2(OE-5) retain fewer eggs *in utero* than N2 animals (p<0.005; p<0.0001, respectively) and *kcnl-2*(tm1885) animals (p<0.0001) (Student’s *t* test). C. Egg-staging assays show that the proportions of young eggs from KCNL-2(OE-2) (54.0±4.3%), KCNL-2(OE-4) (46.0±3.9%), and KCNL-2(OE-5) (46.6±6.7%) are significantly greater than those of N2 (7.2±1.3%) & *kcnl-2*(tm1885) worms (1.6±1.6%) (p<0.05; n=3, Kruskal–Wallis *H* test).

To determine the role of KCNL-2 in the Egl phenotype seen in *kcnl-2*(*tm1885*) animals, we crossed *kcnl-2*(*tm1885*) worms with N2 males to create heterozygotes that were assayed for the number of eggs retained *in utero*. As shown in Figure 6A, *kcnl-2*(*tm1885*) animals consistently showed an increased number of eggs retained *in utero* relative to N2 animals. Heterozygotes retained an average number of eggs that was significantly greater than that of N2 animals, but significantly less than the average number of eggs found in the null strain. Therefore, the mild Egl phenotype demonstrated by *kcnl-2*(*tm1885*) animals was partially rescued in the heterozygotes. Additionally, we transformed the null strain with 1 ng/µl, 10 ng/µl or 90 ng/µl of *p*_*kcnl-2*_ *kcnl-2*(*taa2*)*::gfp* to generate strains KCNL-2(OE-3) (VK2220 and VK2221), KCNL-2(OE-4) (VK1004-1007), and KCNL-2(OE-5) (VK1041-1043), respectively (Figure 6). The data is representative of one line from each strain that was generated by microinjections and was consistent among lines within a strain. As shown in Figure 6B, the average number of eggs retained *in utero* by KCNL-2(OE-3) was not significantly different from the N2 animals, but was significantly different from the *kcnl-2*(*tm1885*) organisms. These results show that transformation of the null strain with 1 ng/µl *kcnl-2* or crossing the null strain with N2 males rescued the Egl phenotype and supports our hypothesis that the Egl phenotype seen in *kcnl-2*(*tm1885*) organisms occurs specifically due to the loss of KCNL-2. Additionally, transgenic lines created with concentrations as low as 10 ng/µl *p*_*kcnl-2*_ *kcnl-2*(*taa2*)*::gfp* into the *kcnl-2*(*tm1885*) background (KCNL-2(OE-4)) produced the hyperactive egg-laying phenotype where the animals retained fewer eggs *in utero* than the wild-type and *kcnl-2*(*tm1885*) animals. Transforming 90 ng/µl *p*_*kcnl-2*_ *kcnl-2*(*taa2*)*::gfp* in the *kcnl-2*(*tm1885*) background (KCNL-2(OE-5)) created a distribution that is not different from KCNL-2(OE-2). Additionally, KCNL-2(OE-4) and KCNL-2(OE-5) organisms lay a significantly greater proportion of early-stage eggs relative to N2 organisms and therefore these organisms hyperactively lay their eggs (Figure 6C). Since the *kcnl-2*(*tm1885*) organisms demonstrate a post-embryonic development delay that is not specific to the loss of KCNL-2, we carried out egg-counting assays on *kcnl-2*(*ok2818*) organisms. As shown in Figure S2, the *kcnl-2*(*ok2818*) organisms are mildly Egl and the average number of eggs *in utero* in staged adults is significantly greater than that of N2 organisms but is not significantly different from the *kcnl-2*(*tm1885*) organisms. Generation of two opposing phenotypes, an Egl phenotype in the KCNL-2 null strains and a hyperactive egg-laying phenotype in transgenic lines that overexpress KCNL-2, supports a specific role of KCNL-2 in the regulation of the rate of egg-laying.

## Discussion

The human KCNN gene family has been linked to several neurological disorders, but the anatomical complexity of the mammalian nervous system limits studies of these channels in these model organisms. All 302 neurons in the *C. elegans* hermaphrodite have been identified and their development have been traced from the embryo to the adult stage [16]. Therefore, isolated neuronal circuits can be potentially teased apart from a larger network and the functions of its resident proteins can be studied in minimal circuits. *C. elegans* can therefore serve as a practical *in vivo* model system to study the physiology of SK channels.

Utilizing a reverse genetics approach, we investigated the functions of KCNL-2 in a putative null strain, *kcnl-2*(*tm1885*), and in transgenic strains that overexpress the channel. The *tm1885* mutation leads to the formation of a null channel since it results in a premature stop codon before the first transmembrane domain of the channel. These organisms are developmentally delayed and undergo larval arrest (Figure S1A). However, transformation of *kcnl-2*(*tm1885*) organisms with the *kcnl-2* gene does not rescue this phenotype. Several explanations may account for this. It has been demonstrated that a truncated K_Ca_2.3 isoform (SKCa3∆) yields the amino terminus of the channel due to a premature stop codon before the first transmembrane domain and has a dominant negative effect on K_Ca_2.2 currents [22]. Similarly, expression of the amino terminal fragment of human K_Ca_2.2 in Jurkat cells abolishes K_Ca_2.2 currents [23]. Therefore, the expression of the amino terminus of KCNL-2 in *kcnl-2*(*tm1885*) mutants could conceivably suppress other natively expressed SK homologues that show an overlapping expression profile in *C. elegans*. In this case, the phenotype would still be KCNL-2-specific, but cannot necessarily be attributed only to the loss of KCNL-2. Alternatively, there remains a possibility that despite backcrossing the *kcnl-2*(*tm1885*) organisms with the N2 organisms eight times, extraneous mutations from TMP treatment may have remained in the *kcnl-2*(*tm1885*) genome. The fact that the *kcnl-2*(*ok2818*) organisms are not developmentally delayed supports the hypothesis that this phenotype is not exclusively due to the loss of KCNL-2.

Here we provide evidence that KCNL-2, an SK channel homologue, plays a role in regulating the rate of egg-laying. As shown in Figure 5A and Figure S2, both *kcnl-2*(*tm1885*) and *kcnl-2*(*ok2818*) mutants retain a significantly greater number of eggs *in utero* than N2 animals, however, this phenotype is relatively mild. This result is not surprising since mechanisms may exist to compensate for the loss of KCNL-2 by upregulating other potassium channels that show overlapping expression. Additionally, we show that the Egl phenotype displayed by *kcnl-2*(*tm1885*) organisms can be rescued by transformation of low DNA concentrations of *kcnl-2* and by crossing with N2 males to produce heterozygotes, thereby further supporting the hypothesis that this phenotype occurs specifically due to the loss of KCNL-2. Approximately 50% of each KCNL-2-overexpressing line showed two phenotypes characteristic of hyperactive egg-laying mutants: they retained fewer eggs *in utero* and greater than 50% of their newly laid eggs were early-stage eggs (Figure 5). The brood size of KCNL-2(OE-2) was not significantly different from that of N2 animals (Figure 2D), supporting the notion that there are no defects in fertility or gametogenesis otherwise.

The egg-laying apparatus consists of two HSNs, six VC neurons and sixteen egg-laying muscles, where the HSN and VC neurons are the only neurons that have been shown to synapse with the vulval muscles [16]. Defects in the egg laying apparatus can cause egg-laying defective or hyperactive egg-laying phenotypes. The HSNs stimulate egg-laying through the neurotransmitter serotonin by causing contraction of the 16 egg-laying muscle cells which leads to expulsion of eggs from the vulva [19,24]. However, the role of the VC neurons remains disputed as they have been shown to have dual functions of inhibition and stimulation of egg-laying [25–27]. Bany and colleagues showed that morphological defects and defects for the synthesis and packaging of acetylcholine in the VC neurons were the underlying basis for hyperactive egg-laying in nine mutants [25]. Hyperactive egg-laying was ultimately due to the lack of inhibition of egg-laying [25]. The hyperactive egg-laying phenotype observed in lines that overexpress KCNL-2 is reminiscent of that obtained when the VC4 & VC5 neurons were laser-ablated; that is, mutant animals that lacked VC4 & VC5 were shown to have a strongly hyperactive egg-laying phenotype where greater than 50% early stage eggs were laid by these organisms [25]. Given that mammalian SK channels function to cause the slow and medium afterhyperpolarization in neurons and that the KCNL-2-b and -c isoforms are expressed in the VC4 and VC5 neurons, we reasoned that knockout of KCNL-2 would increase the excitability of the VC neurons, thereby increasing inhibition and slowing egg-laying [28–30]. Conversely, overexpression would largely decrease the excitability of the VC neurons, slowing inhibition of egg-laying and ultimately leading to a hyperactive egg-laying phenotype. To define the mechanisms by which overexpression of KCNL-2 causes a constitutive egg-laying phenotype, we subcloned the *kcnl-2aii* cDNA and the region of the gene that encodes the *kcnl-2-b* and *-c* splice variants downstream of a VC-specific promoter, pMD64 [25]. Overexpression of this construct in the VC neurons did not reproduce the hyperactive egg-laying phenotype (data not shown). Therefore, it is unlikely that the KCNL-2 channel functions in the VC neurons to produce this phenotype. Alternatively, given that *p*_*kcnl-2*(*atg1*)_ *gfp* and *p*_*kcnl-2*(*atg2*)_ *gfp* are expressed in different neurons and therefore, the KCNL-2 isoforms are very likely to be differentially expressed, it is possible that this phenotype occurs specifically due to overexpression of specific isoforms. However, this possibility was not further tested. Additionally, the *p*_*kcnl-2*(*atg1*)_ *gfp* construct shows expression in the vulval muscles. However, we postulate that KCNL-2 does not function to regulate the rate of egg-laying through the vulval muscles since overexpression of KCNL-2 would hyperpolarize the vulval muscles, decrease the contractility and cause an egg-laying defective phenotype, the opposite of the egg-laying constitutive phenotype seen in the KCNL-2-overexpressing lines. Additionally, none of the *kcnl-2::gfp* fusion constructs showed observable expression in the vulval muscles, suggesting a negligible role in regulating the rate of egg-laying through expression in the vulval muscles.

In a genetic screen, KCNL-2 was identified as a modifier gene of the *C. elegans* survival of motor neuron (SMN) protein [31]. KCNL-2 was shown to affect two of the resulting phenotypes from SMN loss-of-function (*Cesmn-1*) organisms [31]. Knockdown of KCNL-2 by RNAi in *Cesmn-1* mutants proved to enhance growth defects and to decrease pharyngeal pumping defects [31]. As shown in Figure 3B and 3E, KCNL-2 is expressed in the somatic and pharyngeal nervous systems of *C. elegans* and therefore a function for KCNL-2 in pharyngeal pumping is highly conceivable. However, our attempts at RNAi knockdown of KCNL-2 were unsuccessful as RNAi treatment did not result in diminished *p*_*kcnl-2*_ *kcnl-2::gfp* expression in the neurons. The disparity in the RNAi results may have occurred since Dimitriadi et al. used strains that had wild-type copies of the channel, while we measured RNAi knockdown in transgenic lines that overexpressed the KCNL-2 channel [31]. As noted by other investigators, RNAi-sensitive *C. elegans* mutant strains often exhibit abnormalities in their behavior. For example, the *rrf-3* strain has a decreased brood size which could hinder our studies of the egg-laying defects that occur as a function of KCNL-2 expression [32]. Alternatively, the *p*_*unc-119*_ *sid1* strain was transformed with *p*_*kcnl-2*_ *kcnl-2::gfp*, but we found that we did not get efficient RNAi knockdown in these transgenic strains [33]. Since RNAi in neurons is known to be inefficient and since loss of KCNL-2 leads to an Egl phenotype, while overexpression of KCNL-2 causes an Egl-C phenotype, we did not further pursue RNAi experiments.

As delineated by White and colleagues, in addition to the VC neurons, the posterior touch receptor (PLM) neurons and the PVNR form chemical synapses with the HSNs [16]. Inhibition of egg-laying occurs through the ALM and PLM touch receptor neurons which alter the activity of the HSNs [27,34]. Additionally, neuropeptides released by the BAG neurons have been shown to inhibit egg-laying by stimulating EGL-6 which is expressed in the HSNs [35]. Therefore, regulation of the rate of egg-laying is multi-faceted and can be limited by neurons outside the perimeter of the egg-laying circuitry. Given the function of SK channels to regulate the excitability of cells and that KCNL-2 is vastly expressed in neuronal processes that innervate the vulva of the worm, our data supports a role of KCNL-2 in regulating the rate of egg-laying through its expression in neurons that inhibit egg-laying. However, the identity of these neurons remains to be determined.

## Materials & Methods

### Nematode culture and strains

Worms were cultured according to standardized protocols described by Brenner [36]. Worms were grown on nematode growth media (NGM) plates seeded with the OP 50 strain of *E. coli* at standard room temperature unless otherwise noted. The Bristol N2 strain was used. A KCNL-2 mutant strain, *kcnl-2*(*tm1885*), was obtained from Shohei Mitani (National Bioresource Project, Japan). The *kcnl-2*(*tm1885*) strain was outcrossed eight times with the N2 strain to remove extraneous mutations induced by 4,5',8-trimethylpsoralen (TMP) treatment. The *kcnl-2*(*ok2818*) strain was obtained from the CGC and was outcrossed four times with N2 organisms. N2 males were mated with *kcnl-2*(*tm1885*) L4 animals for 24-30 hours, which were subsequently transferred to individual plates and allowed to lay eggs for 24 hours. If ~50% of the progeny were males, then the cross was considered to be successful and the first generation hermaphrodites were used as heterozygotes for the egg-laying assays.

### Transgenic lines

We obtained the WRM063DE08 fosmid from Donald G. Moerman at the *C. elegans* Reverse Genetics Core Facility (Vancouver, Canada). The KCNL-2 channel (Accession number NC_003279) was amplified from this fosmid using 3 pairs of primers: 5’ CTAGCGGGTACCATGTCAAGCTCTGCCCCTTTC3’ and 5’ CGGCGTAGTGATCCAATACGAGAG3’; 5’ CAAGCATAATCTCGCTAGTCC3’ and 5’ CGAGCGGAGTGGTTGAGATGG3’; 5’ ATTCTGTTCGAACTGTCGCCCAG3’ and 5’ CGGATCGCGGCCGCATAGATTATTAAGAACGCCTAG3’ (Integrated DNA Technologies, Coralville, Iowa.) The first amplified fragment was 5,770 bp in length and included 1,951 bp of the promoter region, the second fragment was 5,774 bp in length, and a third fragment of the gene was 3,294 bp in length. Fragments 1, 2 and 3 were digested with KpnI and SalI, SalI and BglII, and BglII and NotI, respectively. These three fragments were sequentially subcloned into the pCR®-Blunt II-Topo® vector (Invitrogen, Carlsbad, CA) to re-construct the full-length gene. The genomic DNA that encodes the KCNL-2 channel displays two initiation sites and two stop codons which allows for alternative splicing and the formation of up to 6 isoforms. GFP was PCR-amplified from the pPD95.75 vector (a gift from A. Fire, Stanford University). Two unique restriction sites, MluI and SacII, were introduced at the initiation sites or stop codons of the open reading frame of KCNL-2. GFP was subcloned between these sites so that the channel was effectively tagged at the amino or carboxy termini. When cloned at the carboxy terminus, the initiation codon was deleted from the GFP coding sequence, ensuring that GFP was not expressed independently of the channel. These constructs have been labeled *p*_*kcnl-2*_ *gfp*:*:*(*atg1*) *kcnl-2*, *p*_*kcnl-2*_ *gfp*:*:*(*atg2*) *kcnl-2*, *p*_*kcnl-2*_ *kcnl-2*(*taa1*)*::gfp* and *p*_*kcnl-2*_ *kcnl-2*(*taa2*)*::gfp* for DNA that was tagged with GFP at the first or second initiation/stop codons, respectively. Transgenic lines were created by micro-injections and those that were used in phenotypic assays are referred to as KCNL-2(OE-1) or KCNL-2(OE-2) when 90ng/µl of *p*_*kcnl-2*_ *kcnl-2*(*taa1*)*::gfp* or *p*_*kcnl-2*_ *kcnl-2*(*taa2*)*::gfp* was transformed in the N2 background or KCNL-2(OE-3), KCNL-2(OE-4) and KCNL-2(OE-5) when 1 ng/µl, 10 ng/µl or 90 ng/µl of *p*_*kcnl-2*_ *kcnl-2*(*taa2*)*::gfp* was transformed in the *kcnl-2*(*tm1885*) background. The transgenic lines used in this study are described in Table S1. From microinjections, four independent KCNL-2(OE-1) and KCNL-2(OE-2) transgenic lines were isolated, while two KCNL-2(OE-3), four KCNL-2(OE-4) and three KCNL-2(OE-5) independent transgenic lines were isolated. Phenotypic assays were carried out on each of these transgenic lines. The data was consistent among the transgenic lines within each KCNL-2-overexpressing strain and therefore the graph represents data from a representative KCNL-2(OE-1), KCNL-2(OE-2), KCNL-2(OE-3), KCNL-2(OE-4) and KCNL-2(OE-5) transgenic line (line a). For imaging, the constructs *p*_*kcnl-2*_ *gfp*:*:*(*atg2*) *kcnl-2* and *p*_*kcnl-2*_ *kcnl-2*(*taa2*)*::gfp* were transformed in the N2 background at 50 ng/µl with an equal concentration of the co-injection marker pRF4. Additionally, two constructs were created to monitor GFP expression downstream of the KCNL-2 promoter. The GFP coding sequence was cloned downstream of the promoter region of KCNL-2, which was composed of 1,951 bp upstream of the first initiation site (*p*_*kcnl-2(atg1)*_ *gfp*). The GFP coding sequence was cloned at the second initiation site where the coding sequence for the KCNL-2-b and -c clones were deleted (*p*_*kcnl-2(atg2)*_ *gfp*). Transgenic lines were created using methods described previously [37]. As summarized in Table S1, the described constructs were injected at 90 ng/µl with 10 ng/µl of the co-injection marker *p*_*myo-2*_ *::mcherry* into N2 and *kcnl-2*(*tm1885*) worms unless otherwise stated.

We obtained the cDNA clones yk1015a02, yk1103e07, yk1295d10 and yk1401h05 from Yuji Kohara (National Institute of Genetics of Japan). The cDNA clones were digested from the parent plasmid pME18S-FL3 with EcoRI and NotI and subcloned in the pcDNA3.1 vector (Invitrogen). All DNA was sequenced with the ABI 3730*xl* DNA Analyzer (University of Pittsburgh, PA).

### Behavioral assays

For all experiments, data analysis is shown for one *kcnl-2*(*tm1885*) strain and two transgenic lines that overexpress KCNL-2 in the N2 background unless otherwise noted. All values were reported as Mean ± SEM and if p<0.05, then the data sets were interpreted as being significantly different. Longevity assays were performed as described previously [38]. Briefly, for each worm strain, the percentage survival of a population of late L4 worms was monitored and recorded daily. The data was plotted on Kaplan-Meier survival curves and analyzed using a Logrank test. The brood size assay was performed as described previously by Singson and colleagues [39]. Briefly, individual late L4 worms were placed onto NGM plates on sequential days until the fertility period came to an end. The number of eggs laid over a five day period by a single subject was measured. The data was analyzed using a Student’s *t*-test. For post-embryonic development (PED) assays (Figure S1), a population of young adult worms (24 hrs post late L4 stage) was allowed to lay eggs for 2 hours, after which the adults were removed and the developmental stage of the progeny was scored after 72 hrs [40]. Normalized values were analyzed using a Kruskal–Wallis *H* test.

### Egg-laying Assays

Koelle & Horvitz have described two methods to assay the rate of egg-laying [19]. The first determines the number of unlaid eggs retained *in utero* per adult animal and the second determines the developmental stage of newly laid eggs. For both egg-laying assays, late L4 animals were grown for 36 hours at 20^o^C to yield a synchronized population of adults. The unlaid egg assay was carried out on eight independent transgenic lines (KCNL-2(OE-1) and KCNL-2(OE-2), lines a to d). To count the number of eggs *in utero*, a total of 30 animals were analyzed per individual transgenic line. Individual animals were dissolved in 1.8% sodium hypochlorite in a Nunc 384 well plate (ThermoScientific, Rochester, NY) and the number of eggs *in utero* was counted after the cuticle dissolved using an inverted microscope. Given that the transgenic KCNL-2-overexpressing lines were not integrated, these strains do not constitute a homogenous population due to the loss of extrachromosomal arrays or genetic mosaicism. Therefore for each experiment, 50% of a synchronized population of adult animals from the KCNL-2(OE-1), KCNL-2(OE-2), KCNL-2(OE-4) and KCNL-2(OE-5) transgenic lines with single rows of eggs (n=25) were selected using a microscope for egg-staging assays. These animals were allowed to lay eggs for 30 minutes on NGM plates with OP 50 as the food source and the eggs were scored as early if they were composed of eight cells or less or late if they had greater than eight cells [35].

### Imaging

Transgenic and mutant animals were immobilized using 6 µl 0.05mM sodium azide in 35 mm optical glass bottomed dishes (MatTek Corporation, Ashland, MA). Widefield images were collected using a standard fluorescence inverted microscope over 40 Z-planes using a 20x 0.75 NA or a 60x 1.4 NA oil Apochromat objective. Epifluorescence was captured using FITC (Ex 488 nm; Em 512 nm) filter sets (Chroma) with a Coolsnap HQ2 CCD camera (Photometrics). Confocal images were collected using a Leica TCS SP8 microscope. GFP fluorescence was illuminated using a 488nm argon laser line with a 63x 1.4NA oil Apochromat CS2 objective. Fluorescence was captured using a spectral HyD detector over ~100 Z-planes. Both widefield and confocal images were visualized, rendered and analyzed using Volocity Visualization Software (v 5.4, PerkinElmer).

## Supporting Information

Figure S1 **Post-embryonic developmental stages of A) N2 *vs. kcnl-2*(tm1885) (n=3; adults: p<0.05; L4: p>0.05; L1-L3 larvae: p<0.05, Kruskal–Wallis *H* test), B) N2, *kcnl-2*(tm1885) and *kcnl-2*(ok2818) or C) N2 vs. KCNL-2(OE-2) (n=3; adults, L4, L1-L3 larvae: p>0.05 ; Kruskal–Wallis *H* test).** The post-embryonic development assay was carried out by allowing a population of 25 young adult worms (24 hrs post late L4 stage) to lay eggs for 2 hours, after which the adults were removed and the developmental stages of the progeny were scored after 72 hrs. Normalized values were analyzed using a Kruskal–Wallis *H* test.
(TIF)

Figure S2 **Unlaid egg assays revealed that the average number of eggs retained *in utero* in *kcnl-2*(*tm1885*) and *kcnl-2*(*ok2818*) are not significantly different, while both strains have a significantly increased number of eggs retained *in utero* relative to N2 organisms (Student’s *t* test, p<0.05).**
(TIF)

Table S1 **List of *C. elegans* strains used in this study.**
(DOCX)
