# Supplementary figures and images for: A Small Conductance Calcium-Activated K+ Channel in C. elegans, KCNL-2, Plays a Role in the Regulation of the Rate of Egg-Laying
Source: PLoS One. 2013 Sep 10;8(9):e75869. doi: 10.1371/journal.pone.0075869 (PMC3769271; doi:10.1371/journal.pone.0075869)

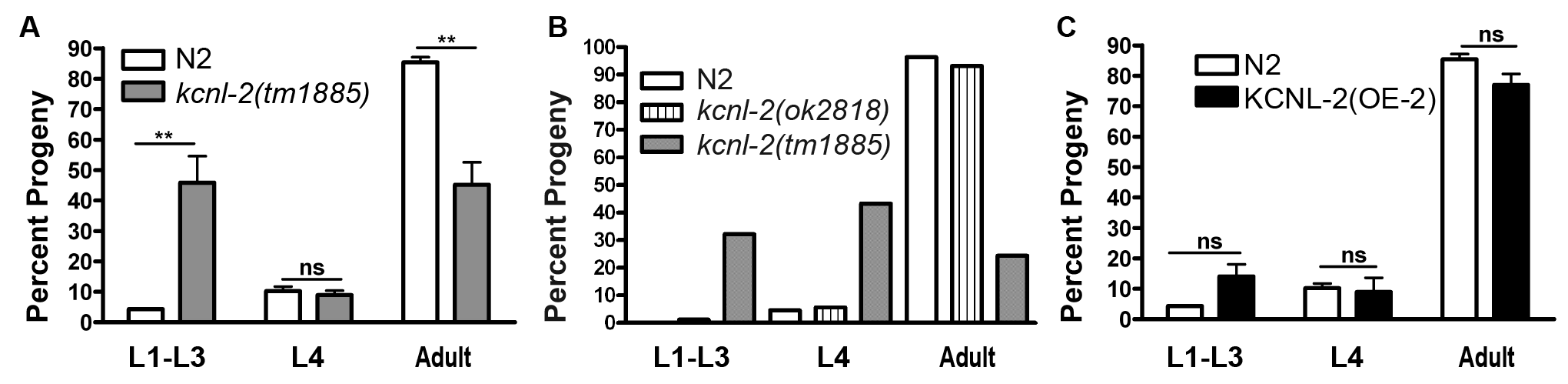

Supplement: Figure S1 — Post-embryonic developmental stages of A) N2 vs. kcnl-2(tm1885) (n=3; adults: p<0.05; L4: p>0.05; L1-L3 larvae: p<0.05, Kruskal–Wallis H test), B) N2, kcnl-2(tm1885) and kcnl-2(ok2818) or C) N2 vs. KCNL-2(OE-2) (n=3; adults, L4, L1-L3 larvae: p>0.05 ; Kruskal–Wallis H test). The post-embryonic development assay was carried out by allowing a population of 25 young adult worms (24 hrs post late L4 stage) to lay eggs for 2 hours, after which the adults were removed and the developmental stages of the progeny were scored after 72 hrs. Normalized values were analyzed using a Kruskal–Wallis H test. (TIF) [file pone.0075869.s001.tif]

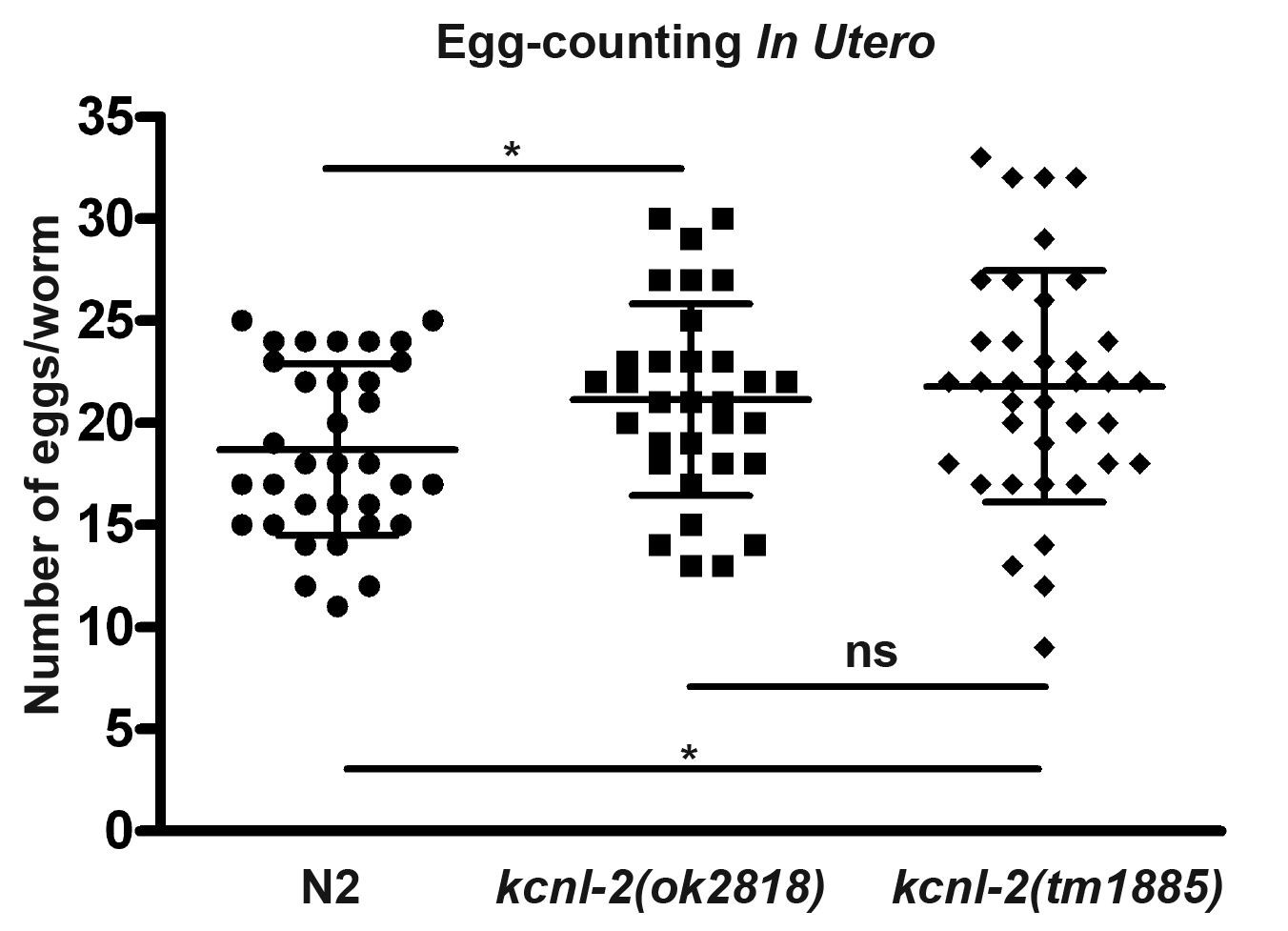

Supplement: Figure S2 — Unlaid egg assays revealed that the average number of eggs retained in utero in kcnl-2(tm1885) and kcnl-2(ok2818) are not significantly different, while both strains have a significantly increased number of eggs retained in utero relative to N2 organisms (Student’s t test, p<0.05). (TIF) [file pone.0075869.s002.tif]
