# Supplementary material for: A Small Conductance Calcium-Activated K+ Channel in C. elegans, KCNL-2, Plays a Role in the Regulation of the Rate of Egg-Laying
Source: PLoS One. 2013 Sep 10;8(9):e75869. doi: 10.1371/journal.pone.0075869 (PMC3769271; doi:10.1371/journal.pone.0075869)
Supplement: Table S1 — List of C. elegans strains used in this study. (DOCX) [file pone.0075869.s003.docx]

| **Strain Name** | **Abbreviation** | **Genotype** | **Concentration**  **transformed** |
| --- | --- | --- | --- |
| VK1000 | KCNL-2  (OE-1) | *N2;vkEx1000[Pkcnl-2kcnl-2TAA1]::GFP Line A* | *90 ng/μl* |
| VK1001 |  | *N2;vkEx1001[Pkcnl-2kcnl-2TAA1]::GFP Line B* |  |
| VK1002 |  | *N2;vkEx1002[Pkcnl-2kcnl-2TAA1]::GFP Line C* |  |
| VK1003 |  | *N2;vkEx1003[Pkcnl-2kcnl-2TAA1]::GFP Line D* |  |
| VK1065 | KCNL-2  (OE-2) | *N2;vkEx1065[Pkcnl-2kcnl-2TAA2]::GFP Line A* | *90 ng/μl* |
| VK1066 |  | *N2;vkEx1066[Pkcnl-2kcnl-2TAA2::GFP] Line B* |  |
| VK1067 |  | *N2;vkEx1067[Pkcnl-2kcnl-2TAA2::GFP] Line C* |  |
| VK1068 |  | *N2;vkEx1068[Pkcnl-2kcnl-2TAA2::GFP] Line D* |  |
| VK2220 | KCNL-2  (OE-3) | *kcnl-2(tm1885);vkEx2220[Pkcnl-2kcnl-2TAA2::GFP] Line A* | *1 ng/μl* |
| VK2221 |  | *kcnl-2(tm1885);vkEx2221[Pkcnl-2kcnl-2TAA2::GFP] Line B* |  |
| VK1004 | KCNL-2  (OE-4) | *kcnl-2(tm1885);vkEx1004[Pkcnl-2kcnl-2TAA2::GFP] Line A* | *10 ng/μl* |
| VK1005 |  | *kcnl-2(tm1885);vkEx1005[Pkcnl-2kcnl-2TAA2::GFP] Line B* |  |
| VK1006 |  | *kcnl-2(tm1885);vkEx1006[Pkcnl-2kcnl-2TAA2::GFP] Line C* |  |
| VK1007 |  | *kcnl-2(tm1885);vkEx1007[Pkcnl-2kcnl-2TAA2::GFP] Line D* |  |
| VK1041 | KCNL-2  (OE-5) | *kcnl-2(tm1885);vkEx1041[Pkcnl-2kcnl-2TAA2::GFP] Line A* | *90 ng/μl* |
| VK1042 |  | *kcnl-2(tm1885);vkEx1042[Pkcnl-2kcnl-2TAA2::GFP] Line B* |  |
| VK1043 |  | *kcnl-2(tm1885);vkEx1043[Pkcnl-2kcnl-2TAA2::GFP] Line C* |  |
| VK1323 | *--* | *N2; vkEx1323[p_kcnl-2(atg1)_gfp] Line A* | *50 ng/μl* |
| VK1327 | -- | *N2; vkEx1327[p_kcnl-2(atg2)_gfp] Line A* | *50 ng/μl* |
| VK1567 | -- | *N2;vkEx1567[p_kcnl-2_kcnl-2(taa2)::gfp] Line A* | *50 ng/μl* |
| VK1401 | *--* | *N2;vkEx1401[p_kcnl-2_gfp::(atg2)kcnl-2] Line A* | *50 ng/μl* |
